# The data do not seem to support a benefit to BCAA supplementation during periods of caloric restriction

**DOI:** 10.1186/s12970-016-0128-9

**Published:** 2016-05-11

**Authors:** Brad P. Dieter, Brad Jon Schoenfeld, Alan A. Aragon

**Affiliations:** Providence Medical Research Center, Providence Sacred Heart Medical Center and Children’s Hospital, Research Discovery Lab, Spokane, WA USA; Department of Health Sciences, Lehman College, Bronx, NY USA; AARR, Northridge, CA USA

## Abstract

J Int Soc Sports Nutr 13:1-015-0112-9, 2016 describe the efficacy of branched chain amino acid (BCAA) supplementation and resistance training for maintaining lean body mass during a calorie-restricted diet, and claim that this occurs with concurrent losses in fat mass. However, the reported results appear to be at odds with the data presented on changes in fat mass. This letter discusses the issues with the paper.

To the Editor,

Dudgeon et al. [1] describe the efficacy of branched chain amino acid (BCAA) supplementation and resistance training for maintaining lean body mass during a calorie-restricted diet, and claim that this occurs with concurrent losses in fat mass. However, the reported results appear to be at odds with the data presented on changes in fat mass.

The study reports a statistically significant change in fat mass for the group supplementing with BCAAs, but not in the placebo (isocalorically matched carbohydrate [CHO] beverage) group. However, this outcome is paradoxical with the results. Table 2 states that the BCAA group lost 0.6 kg of fat mass while the CHO group lost 1.4 kg. Given that the standard errors were virtually identical between groups (the SE was actually less for the CHO group pre-study), it is counterintuitive to believe that statistical probability for a true effect would be higher in the group that supplemented with BCAA.

**Table 2:** Changes in body mass variables before and after 8 week study period

	Age (yrs)	Height (cm)	Body Mass (kg)	Lean Mass (kg)	Fat Mass (kg)
BCAA	24.7 ± 0.6	177.9 ± 4.6	84.3 ± 5.2	72.2 ± 4.7	12.2 ± 0.7
			84.2 ± 4.8	72.6 ± 4.3	11.6 ± 0.7^a^
CHO	23.5 ± 0.6	176.6 ± 5.6	78.3 ± 2.9	67.8 ± 2.5	105 ± 0.5
			76.0 ± 2.4^a^	66.9 ± 2.5^a^	9.1 ± 0.7

^a^denotes significant difference (*p* < 0.05) within BCAA and CHO

All subjects were prescribed the same hypocaloric diet and exercise programs. The BCAA group received 28 g of BCAA (14 g prior/during each workout and 14 g post workout) while the CHO group received 28 g of a carbohydrate/electrolyte supplement (14 g prior/during each workout and 14 g post workout)

If the results were in fact correctly reported with respect to statistical probability, then this appears to be a case of using the wrong statistical measures to analyze the data. The authors quizzically chose a combination of paired and unpaired t-tests for analysis, which is prone to bias based on the uniformity of the direction of change for each subject. Indeed, multiple t-tests heighten the probability of making at least one type one error, and the increased error rate may be substantial [2]. The appropriate statistical measures that should have been employed include repeated measures ANOVA, repeated measures mixed model, or perhaps an ANCOVA on the change scores. Moreover, a priori alpha values alone provide an incomplete picture of the importance of results [3]. Magnitude-based statistics such as effect size (ES) are necessary to provide a more comprehensive perspective of the relative meaningfulness of the results and thus draw appropriate practical implications from findings [4]. A computation of Cohen’s D based on the data shows that the ES for change in fat mass for CHO condition was strong (0.81) while that of the BCAA condition was weak (0.29). In addition, given the small sample size it would have been beneficial to present the individual data points to show changes in fat mass and lean mass over the course of the study instead of the aggregate data. Lastly, given the discordance between statistical significance and physiological meaningfulness power calculations ought to have been conducted to indicate that the failure to reject the null hypothesis in the CHO group may have been a type II error.

Issues with interpreting results are further confounded by inconsistencies in the reported data. In the abstract and the results section the authors indicate that the BCAA group lost 0.05 kg ± 0.08 kg. We were unable to locate this data anywhere else in the manuscript. Based upon the data in Fig. 1 (Fig. 4 from the original paper), it appears that the mean decrease in fat mass was actually 0.5 %, raising the possibility of an error in transcription. Another glaring issue with the data are the standard error bars, which do not seem to match the data in the text. For example, the BCAA group appears to be roughly 0.4 kg, instead of the 0.08 kg reported in the text and the 0.08 kg put forth in the abstract. These discrepancies call into question the veracity of the study.

**Fig. 1:**
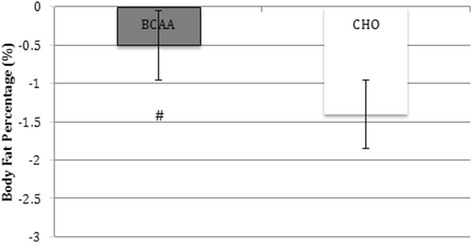
Fat Mass Change

Finally, the data presented in the paper appear to be internally inconsistent. The resting metabolic rate (RMR) dropped significantly in the BCAA group (412 kcal/day) but not in the CHO group (no data presented). In the discussion, the authors quote, “The amount of lean tissue mass is essential in determining metabolic rate, where a greater amount of lean tissue increases RMR.” Given the maintenance of lean body mass and the loss of fat mass in the BCAA group, and the loss of lean body mass and the apparent loss of fat mass in the CHO group, the RMR should have decreased in the CHO group but not the BCAA group. These phenomena ought to have been explained by the authors as it conflicts with the reported outcome of maintenance of lean body mass and greater fat loss in the BCAA group while the CHO group lost lean body mass.

When attempting to extrapolate the findings into practice, it would appear that changes in body composition are a product of the magnitude of weight loss as opposed to the supplementation protocol. Specifically, the greater preservation of LBM in the experimental condition can be attributed to the minimal loss of body fat in these subjects, not consumption of BCAA. On the other hand, the control group lost substantially more weight, so it would seem logical that they would not have retained LBM as well. The alternative hypothesis posed by the authors is not consistent with the current body of literature. As noted in a recent review by Morton et al. [5], there is a paucity of evidence supporting a beneficial effect for BCAA supplementation in promoting increases in muscle protein synthesis or lean mass, and in fact there might be a detrimental impact given that the AAs appear to antagonize each other in terms of transport both into circulation and likely into the muscle.

**Competing interests**

The authors declare that they have no competing interests.

**Authors’ contributions**

All authors drafted the text, read and approved the final manuscript.
