# Supplementary material for: The data do not seem to support a benefit to BCAA supplementation during periods of caloric restriction
Source: J Int Soc Sports Nutr. 2016 May 11;13:21. doi: 10.1186/s12970-016-0128-9 (PMC4865017; doi:10.1186/s12970-016-0128-9)
Supplement: Related texts

# Reply: Authors’ response

**Authors:** Brad P. Dieter, Brad Jon Schoenfeld, Alan A. Aragon

The authors would like to thank JISSN for the opportunity to respond to the referenced communication and we appreciate the critical review of our work by Schoenfeld et. el. In the correspondence we will address what we feel to be the primary issues raised by Schoenfeld et. al.

First, in regards to which statistical method was used, the traditional mechanism for testing a pre vs. post treatment is *t*-test (paired for within groups and unpaired for between groups). Choosing a statistical methodology, regardless of type, will lend itself to either Type I or Type II error. This is not a debatable point. In choosing to answer our research question, “Does BCAA supplementation impact lean mass while on a hypocaloric diet?” we chose, and the JISSN editors and reviewers each independently agreed, a proven statistical model that has been used innumerable times in the literature when pre vs. post analysis of a treatment is examined.

Next, we have chosen to include a spaghetti graph of the body composition data, both lean tissue mass and fat mass, to allow the readers to see how each of the individuals responded during our intervention. As can be seen in Fig. 2 (Fig. 3 from the original paper), all control (CHO) subjects lost lean tissue mass, while in the BCAA group three gained and six lost lean tissue mass. Concerning fat mass, four CON subjects gained and four CON subjects lost, while all the BCAA group lost fat mass. (See Fig. 3 (Fig. 2 from the original paper)) Further, it should be clarified that Fig. 1 (Fig. 4 from the original paper) in the initial manuscript should have been labeled “Change in Percent Body Fat” not “Fat Mass Change.” The corrected graph can be seen below (Fig. 4 (Fig. 1 from the original paper)).

**Fig. 2:** Individualized changes in lean tissue mass following eight-week study period as determined by hydrostatic weighing. BCAA group received BCAA product (14 g prior/during each workout and 14 g post workout) while the control group received 28 g carbohydrate/electrolyte mixture at the same times. All subjects followed an individualized hypocaloric diet and resistance training program

**Fig. 3:** Individualized changes in fat mass following eight-week study period as determined by hydrostatic weighing. BCAA group received BCAA product (14 g prior/during each workout and 14 g post workout) while the control group received 28 g carbohydrate/electrolyte mixture at the same times. All subjects followed an individualized hypocaloric diet and resistance training program

**Fig. 4:** Change in body mass following eight-week study period as determined by hydrostatic weighing. BCAA group received BCAA product (14 g prior/during each workout and 14 g post workout) while the control group received 28 g carbohydrate/electrolyte mixture at the same times. All subjects followed an individualized hypocaloric diet and resistance training program. # denotes significant difference (*p* < 0.05) within BCAA and CHO

Another issue raised by Schoenfeld et. al. was the lack of a change in RMR when changes were found in body composition. The variability in the data did not allow for significant changes in RMR to be found. As noted in our manuscript, we acknowledge that this did not fit given the changes detected in body mass. If more subjects were to have been involved in the study, perhaps a clearer picture of the impact of BCAA on RMR would have been found.

Finally, all body weight changes are relative to the individual, thus the comparison within groups and not just between. The impact of a hypocaloric diet is being ignored in the correspondence and it should not be since this, as noted in the title, is what we feel makes this a significant contribution to the body of scientific literature. Heavy resistance training without adequate calories to maintain lean mass will inevitably send the body into a catabolic state, as evidenced in this study by the loss in body mass, both fat and lean, in the CHO group. In our study, the addition of a BCAA-containing supplement, which has well documented anabolic effects [6], blunted the loss in lean mass in subjects in a hypocaloric state. This anabolic effect of BCAA’s has been shown in healthy RT males [7] and in persons attempting to lose weight [8, 9], but scarce data exits pertaining to hypocaloric diets.

Is this the seminal study on amino acid supplementation, body mass and resistance training?. By no means. However, what this study does is add to the knowledge base and present other research scientists the opportunity to mimic or expand on our methodology and results to determine what the data collectively says about this specific question. As noted in our acknowledgements, performing heavy resistance exercise while following a strict hypocaloric diet is difficult, to say the least. Thus, there is a paucity of peer-reviewed work looking at reduced caloric intake, amino acid supplementation, body composition and muscle performance.
